# Disparities in radiation therapy delivery: current evidence and future directions in head and neck cancer

**DOI:** 10.1186/s41199-016-0005-x

**Published:** 2016-06-13

**Authors:** Henry S. Park, Roy H. Decker

**Affiliations:** Department of Therapeutic Radiology, Yale University School of Medicine, 15 York St, New Haven, CT 06519 USA

**Keywords:** Disparities, Inequity, Race, Age, Insurance, Socioeconomic, Delivery, Radiation therapy, Radiotherapy, Head and neck cancer

## Abstract

**Background:**

Though treatments for head and neck cancer have improved in recent years, significant variation persists in the delivery of surgery, radiation therapy, and systemic therapy to patients throughout the United States.

**Body:**

In this review, we explore the current evidence regarding radiation therapy utilization inequities across the spectra of race, socioeconomic status, and age. We also discuss hypothesized mechanisms for how non-clinical factors may influence shared clinical decisions between patients and providers. Finally, we suggest future directions for research in treatment disparities.

**Conclusions:**

Radiation therapy continues to be delivered inequitably among certain subpopulations with head and neck cancer and other cancers. More research into the drivers of these disparities and interventions designed to address them are necessary.

## Background

The multidisciplinary management of head and neck cancer (HNC) has advanced rapidly in recent years. Sophisticated conformal radiation therapy techniques like intensity-modulated radiation therapy and proton beam radiation therapy, surgical approaches like transoral robotic surgery, and targeted biologic agents like cetuximab have been increasingly utilized in combination with each other to maximize tumor control while minimizing toxicities. In the setting of the rising prevalence of HPV-associated HNC, much research is devoted to optimizing management strategies for every patient subgroup.

However, not all patients may have equal access to such advancements in cancer therapy, specifically radiation therapy. In this review, we explore the current evidence demonstrating radiation therapy utilization inequities across the spectra of race, socioeconomic status, and age. We first focus on HNC before expanding to evidence from other more common malignancies in order to allow for a broader context. We also discuss hypothesized mechanisms for how sociodemographic factors may influence shared clinical decisions between patients and providers. Finally, we suggest potential future directions for research and interventions in this area.

## Main text

### Evidence of radiation therapy delivery disparities in HNC

One of the most heavily studied subjects in cancer treatment delivery disparity research has revolved around race. Two recent manuscripts have indicated differences in definitive treatment receipt by racial group for patients with HNC. First, Mahal et al. used Surveillance, Epidemiology, and End Results (SEER) database to determine that African-American patients with non-metastatic HNC were less likely to receive definitive treatment (surgery, radiation therapy, or both per National Comprehensive Cancer Network guidelines) than those who were not African-American (adjusted odds ratio 0.63, 95 % confidence interval 0.55–0.72) [1]. These results persisted in subsets of patients with cancers of the oral cavity, hypopharynx, nasopharynx, and larynx, but not oropharynx. In a sensitivity analysis in which other-cause mortality was used as a proxy for comorbidity in multivariable logistic regression, the results did not change significantly. Second, Subramanian et al. also examined the effect of race on treatment receipt among Medicaid patients in California and Georgia with HNC [2]. After adjustment for demographics, stage at diagnosis, and tumor site, black race was not associated with differences in radiotherapy utilization, though it was associated with a lower likelihood of receiving surgery.

Medicaid insurance and lack of insurance have also been associated with disparities in treatment delivery in HNC. Inverso et al. reported that after adjustment for patient demographic data, socioeconomic factors, and tumor characteristics, uninsured patients with nonmetastatic HNC in the SEER database were more likely to not receive definitive treatment than those with any type of insurance (adjusted odds ratio 1.64, 95 % CI 1.37–1.96) [3]. Sensitivity analyses further categorizing insurance status found that patients with no insurance or Medicaid insurance were more likely to not receive definitive treatment than those with private insurance.

Appropriate receipt vs. inappropriate omission of radiation therapy is not the only factor affecting optimal treatment delivery. Prolonged time from cancer diagnosis to treatment initiation may have an impact on tumor control and mortality [4–6]. Murphy et al. noted significant variation in time to treatment initiation by race, Hispanic ethnicity, insurance status, zip-code-level income, zip-code-level education, and age among patients with HNC in the NCDB [6].

Another source of disparities in high-quality radiation therapy delivery may be access to advanced techniques like intensity-modulated radiation therapy or proton beam radiation therapy. Both modalities have been associated with significant improvements in toxicities and quality-of-life [7–9], with one retrospective study even suggesting a cancer-specific survival benefit to intensity-modulated radiation therapy over 3D-conformal radiation therapy [10]. Using the SEER-Medicare linked database, Sher et al. found that patients living in a census tract with higher median income were significantly more likely to be treated with intensity-modulated radiation therapy [11]. No difference in intensity-modulated radiation therapy utilization by race, sex, or age was noted in any of the three published SEER-Medicare studies on this subject [10–12]. The value of proton beam radiation therapy for HNC is currently under active investigation. Though there are no publications available regarding disparities in proton beam radiation therapy utilization in HNC to our knowledge, this is potentially a promising field of study in the future.

An increasing amount of data supports the hypothesis that radiation therapy by high-volume providers is associated with improved outcomes in HNC [13, 14] and multiple non-HNC malignancies like lung, cervical, and prostate cancers [15–18]. In HNC, two recent large national database analyses have noted disparities in access to high-volume providers, which may serve as another proxy for access to high-quality cancer care. First, Boero et al. found that in the SEER-Medicare linked database, white patients receiving 3D-conformal radiation therapy and intensity-modulated radiation therapy for HNC were more significantly likely to be treated by high-volume radiation oncologists than non-white patients [13]. Second, Wuthrick et al. reported that patients with private insurance were more likely to receive HNC treatment at high-accruing facilities into Radiation Therapy Oncology Group trials than those without private insurance [14].

Table 1 summarizes the primary themes of the disparities observed in HNC.

**Table 1:** Summary of major themes associated with disparities in radiation therapy delivery

Disparity Theme	Evidence for Disparities in Head & Neck Cancer
1) Underutilization of Definitive Radiation Therapy and/or Surgery	Race [1, 2]; Insurance [3]
2) Delayed Time from Diagnosis to Radiation Therapy Initiation	Race, Ethnicity, Insurance, Age, Income, Education [6]
3) Underutilization of Advanced Radiation Therapy Techniques (i.e. Intensity-Modulated Radiation Therapy)	Income [11]
4) Limited Access to High-Volume Radiation Therapy Providers	Race [13]; Insurance [14]

### Evidence of radiation therapy delivery disparities in non-HNC malignancies

It should be noted that racial, socioeconomic, and age disparities in radiation therapy delivery are well-documented in non-HNC cancers, such as prostate cancer [19]. Based on SEER data, African-American patients were less likely to receive curative-intent therapy (adjusted odds ratio 0.82, 95 % confidence interval 0.79–0.86), especially among patients with NCCN high-risk disease (adjusted odds ratio 0.60, 95 % confidence interval 0.56–0.64) [20]. Filipino men were also less likely to receive definitive treatment in localized prostate cancer in a SEER study of Asian-Americans [21]. Among patients receiving definitive therapy, African-American men are more likely to receive radiation therapy but less likely to undergo surgery [20, 22].

In addition, Bledsoe et al. examined the effect of insurance status on treatment selection among nonelderly patients with prostate cancer in the NCDB [23]. Even after adjustment for race and other sociodemographic and clinical factors, Medicaid patients were less than half as likely to receive minimally invasive surgery and instead were more than twice as likely to receive external beam radiation therapy compared to patients with private insurance. There were no differences in minimally invasive surgery and external beam radiation therapy utilization rates between patients with Medicaid insurance compared to no insurance at all. For prostate cancer patients who do receive radiation therapy in the National Cancer Data Base (NCDB) database, black and Hispanic patients were found to be significantly less likely to receive proton beam radiation therapy than white patients [24].

There is also evidence of disparities in treatment delivery beyond external beam radiation therapy in HNC and prostate cancer. Grant et al. examined patients in the SEER database to determine the association between insurance status and brachytherapy receipt [25]. The study showed that patients who received radiation therapy definitively for prostate and cervical cancer or postoperatively for breast cancer were less likely to receive brachytherapy if they had Medicaid coverage (odds ratio 0.57, 95 % CI 0.53–0.61) or no insurance coverage (odds ratio 0.50, 95 % CI 0.45–0.56) compared to those with non-Medicaid insurance. A SEER study of 3,851 black patients and 44,010 white patients with rectal cancer showed that black patients were significantly more likely to receive no radiation therapy for stage II to III disease (adjusted odds ratio 1.30, 95 % confidence interval 1.15–1.47) [26]. Older patients were less likely to receive the standard-of-care combination of radiation therapy with fluorouracil-based chemotherapy among 1,807 Medicare beneficiaries with stage II to III rectal cancer in the SEER-Medicare database [27]. Similar trends were noted in stage I to II breast cancer, as older women were less likely to receive optimal local treatment with radiation therapy and surgery [28, 29].

### Potential mechanisms

We have now shown that sociodemographic factors may play a significant role in contributing to gaps in radiation therapy delivery. Disparities in radiation therapy delivery may be at least partially related to differences in referral patterns to radiation oncologists. The lung cancer literature has studied this feature most extensively. Goulart et al. analyzed data from 28,977 patients with stage III and IV non-small cell lung cancer diagnosed in 2000–2005 from the SEER-Medicare database linked with the American Medical Association Masterfile database [30]. On multivariable analysis, older age, black race, and female sex were associated with a lower likelihood of seeing all cancer specialists (medical oncologists, radiation oncologists, and thoracic or general surgeons). Seeing all three types of cancer specialists was predictive of a significantly higher likelihood of receiving National Comprehensive Cancer Network (NCCN) guideline-based therapies. Although these numbers were not explicitly reported in the manuscript, our calculations reveal that the likelihood of receiving guideline-based therapies for patients with vs. without radiation oncologist referral was 64.1 % vs. 20.1 % for stage IIIA disease and 56.3 % vs. 6.3 % for stage IIIB disease.

Australian data by Vinod et al. also show disparities in radiation oncologist referrals [31]. Based on questionnaire data from diagnosing and treating clinicians for 1,812 lung cancer patients from New South Wales, the authors found significant underutilization of curative-intent radiation therapy to the primary site (20 % actual vs. 50 % optimal), especially in patients with limited-stage small cell lung cancer (46 % actual vs. 94 % optimal). Older patients were again less likely to be referred to radiation oncologists, as were patients who lived in areas that were not highly accessible by distance to major service centers based on the Accessibility and Remoteness Index for Australia. Patient sex did not impact referral patterns, while race and income level were not analyzed in this study.

Other reasons for disparities in radiation therapy delivery can be considered within a framework involving three broad categories: poverty, culture, and social injustice [32]. Barriers related to poverty and low socioeconomic status include the lack of a primary care physician, who would conduct screening and diagnostic follow-up; limited access to healthcare based on geographical inconveniences; competing survival priorities such as obtaining food, shelter, and safety; medical comorbidities; lack of adequate health insurance; lack of information and knowledge; and risk-promoting lifestyles, like poor nutrition and physical inactivity [33, 34].

Cultural factors, which reflect a set of learned and shared beliefs, values, traditions, world views, communication styles, and behavior common to a particular social group, can also play a large role in racial disparities in treatment delivery. Factors like spirituality, perceived susceptibility to cancer, cultural beliefs about cancer, and medical mistrust can be major barriers for certain cultural groups [33]. For instance, black women often consider themselves at lower risk for developing breast cancer than white women, even among those with a family history of breast cancer, which may translate into low perceived need for mammography or delays in seeking treatment for a breast abnormality [35]. There may also be a more fatalistic attitude regarding breast cancer treatment, less confidence in Western medicine, more confidence that spirituality and divine intervention are more effective in promoting cure, and a cultural norm against discussing breast cancer among certain cultural groups [36–39]. Traditional practices like Ayurvedic and Traditional Chinese medicine or Mexican herbal mixtures may or may not have beneficial or harmful effects on cancer treatment, especially regarding interactions with radiation therapy or chemotherapy. However, disclosure of this information by patients may be hindered by fear of receiving judgmental or dismissive comments from oncology providers, thereby excluding the potential of communication about these issues [34]. Patient-provider communication is also critical when addressing medical truth-telling at the end-of-life in certain family and community-centered societies, where practices of nondisclosure often persist due to cancer-related social stigma [34].

Social injustice, including factors like racial prejudice and discrimination, may also factor into racial disparities, but this relationship does not appear to have been as well studied as socioeconomic status or culture [32]. Provider perceptions of racial minority patients may affect quality of care, as physicians rated black patients with coronary artery disease as less educated and less likely to comply with treatment, even after adjusting for socioeconomic status [40]. In addition, black women were more likely to report a lack of physician recommendation as a reason for not undergoing mammography [41]. While it is certainly debatable whether or not these findings due to racial prejudice or other factors, perceived racial discrimination by patients may also play a role in differences in cancer incidence as well as treatment and satisfaction with care [42]. For instance, black women younger than 50 years who reported higher levels of racial discrimination in “everyday” experiences were at greater risk of subsequently developing breast cancer, since perceived racism could act as a chronic stressor that alters immune functioning and/or endogenous hormone levels [43].

## Conclusions

Radiation therapy continues to be delivered inequitably among certain subpopulations with head and neck cancer and other cancers. Ultimately, it appears that the key to future research on treatment disparities in cancer lies upon disentangling apparent effects of race, poverty, age, education, and discrimination. It is also important to improve the measurement accuracy of specific indicators of socioeconomic status beyond broad measures of household income in a given zip code or census tract. In order to close these gaps, we must evaluate various communication practices in the way treatments are decided and patient autonomy is upheld.

We must also venture well beyond medical care itself. Patient education regarding high-risk behavior like smoking, obesity, and environmental hazards; programs facilitating travel to healthcare organizations; legislative action to improve access to healthcare; and general improvements in housing, schooling, and neighborhood safety must all be addressed before disparities caused by these factors can be minimized. However, it is still unclear if and how interventions addressing these areas could make a measurable difference.

With improving awareness of the complexity of this problem, there will hopefully be a growth in research infrastructure capturing this necessary data. More sophisticated analyses that account for these covariates will help clarify the most critical determinants of these disparities and to create and evaluate interventions on individual, locoregional, national, and international levels to address and eliminate them.

## Abbreviations

HNC: head and neck cancer
NCDB: National Cancer Data Base
SEER: Surveillance, Epidemiology, and End Results
